# Use of Mammoplasty to Treat a Recurrent Breast Abscess: Improving Cosmetic Outcomes by Incorporating Principles of Oncoplastic Breast Surgery

**DOI:** 10.7759/cureus.94740

**Published:** 2025-10-16

**Authors:** Ceri Gillett, Sima Patel, Ehsanur Rahman

**Affiliations:** 1 Surgery, New Cross Hospital, Wolverhampton, GBR; 2 Surgery, Royal Wolverhampton NHS Trust, Wolverhampton, GBR

**Keywords:** breast surgery, oncoplastic breast surgery, onco reconstruction, onco-surgery, therapeutic mammoplasty

## Abstract

Chronic inflammatory breast disease, including recurrent mastitis, can significantly affect both physical and psychological well-being. Complications such as chronic abscesses and fistula formation often necessitate multiple surgical interventions, resulting in scarring, deformity, and patient distress. Optimal management remains challenging when medical therapy fails.

We present two cases of chronic breast abscesses managed with oncoplastic techniques using bilateral mammoplasty, highlighting the potential for both disease resolution and favourable cosmetic outcomes.

Clinical records were reviewed via the institutional web portal. A targeted literature search was conducted using PubMed. Two patients with recurrent breast abscesses unresponsive to conservative management underwent bilateral mammoplasty. Clinical outcomes, including resolution of infection and aesthetic results, were evaluated.

Both patients achieved complete resolution of chronic abscesses with no recurrence at follow-up. Aesthetic outcomes were satisfactory, with preservation of breast contour and minimal scarring. Patients reported improved psychological well-being and quality of life.

Mammoplasty represents a viable surgical option for selected patients with chronic breast abscesses refractory to conventional management. By incorporating oncoplastic principles, this approach can provide definitive treatment while maintaining favourable cosmetic outcomes. Further prospective studies are warranted to evaluate the efficacy and long-term outcomes of radical surgical interventions in this patient population.

## Introduction

Benign inflammatory breast disease and infection constitute a significant clinical burden, accounting for up to 5% of breast-related hospital admissions within the National Health Service (NHS) [1]. Mastitis, defined as inflammation of the breast tissue, can be broadly categorised into lactational and non-lactational types. Among the latter, granulomatous mastitis represents a distinct but uncommon entity that primarily affects younger women. It is a benign inflammatory condition of uncertain aetiology, yet its variable clinical presentation, chronic course, and propensity for recurrence place a considerable burden on both patients and healthcare services. Patients frequently present with breast pain, palpable tender masses, erythema, overlying skin changes, or chronic abscess formation.

Breast abscesses themselves are characterised by localised collections of purulent material within inflamed breast tissue. First-line management includes antibiotic therapy and ultrasound-guided needle aspiration, with surgical drainage generally reserved for refractory cases in which less invasive measures have failed. Although incision and drainage may provide symptomatic relief, the cosmetic implications, risk of fistula formation, and potential for prolonged recovery limit its use. Chronic or recurrent abscesses often develop following inadequately treated acute abscesses or as a complication of granulomatous mastitis [2]. Such cases may present with persistent pain, discharging sinuses, or repeated inflammatory episodes, occasionally progressing to fistula formation. Fistulae arise as epithelialised tracts that initially contain granulation tissue, which subsequently matures to keratinised epithelium, thereby preventing spontaneous healing [2,3]. This is where the principles of oncoplastic surgery, designed to resect significant volumes of tissue while maintaining or restoring breast form, become highly relevant.

The diagnostic challenge of granulomatous mastitis is heightened by its clinical overlap with malignant breast conditions such as inflammatory carcinoma [2]. Furthermore, repeated surgical intervention is often associated with poor cosmetic outcomes, scarring, deformity, and significant psychological distress [2,4]. While oncoplastic techniques are standard in oncology, their application for complex benign breast conditions remains underreported and represents a potential paradigm shift in management.

In this context, alternative surgical strategies that offer both definitive treatment and preservation of breast aesthetics warrant exploration. We present two cases of recurrent breast abscesses in which medical therapy was unsuccessful, and surgical management using mammoplasty was undertaken to achieve disease control while maintaining favourable cosmetic outcomes.

## Case presentation

Case 1

**Figure 1:**
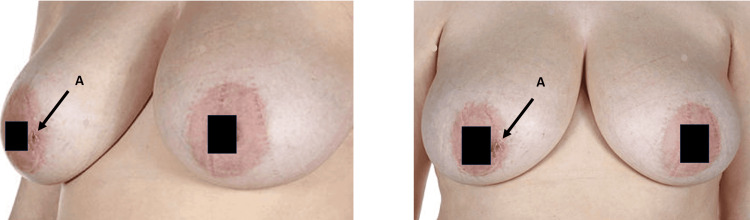
Preoperative images with right breast fistula as indicated by label A

We present the case of a 41-year-old female patient with a four-year history of intermittent pain, inflammatory changes, and discharge from the areola complex. The patient had no significant comorbidities and was a non-smoker. She had previously undergone repeated Hadfield’s procedures in 2008 and 2012, which provided minimal symptomatic relief. Due to recurrent fistula formation and the substantial negative impact on her quality of life, a decision was made to pursue a more definitive surgical approach. In 2016, the patient underwent excision of the right mammary fistula combined with bilateral reduction mammoplasty using the inferior pedicle. The objectives of surgery were twofold: to reduce the risk of disease recurrence and to improve quality of life, which had been significantly compromised by chronic pain and persistent discharge following multiple prior procedures. Follow-up at six, 12, and 24 months demonstrated complete resolution of symptoms, no disease recurrence, and satisfactory cosmetic outcomes. Figure 1 shows preoperative images with a right breast fistula.

**Figure 2:**
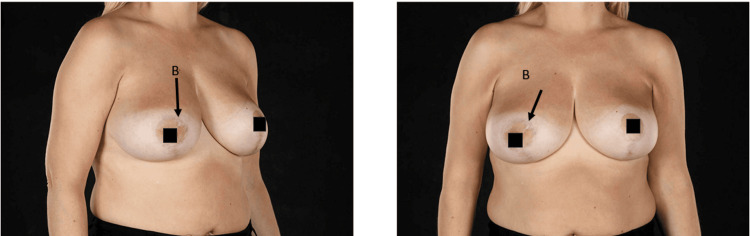
Post-operative images taken one year after surgery. The scar is indicated by label B.

Figure 2 shows post-operative outcomes one year post surgical intervention.

Case 2

**Figure 3:**
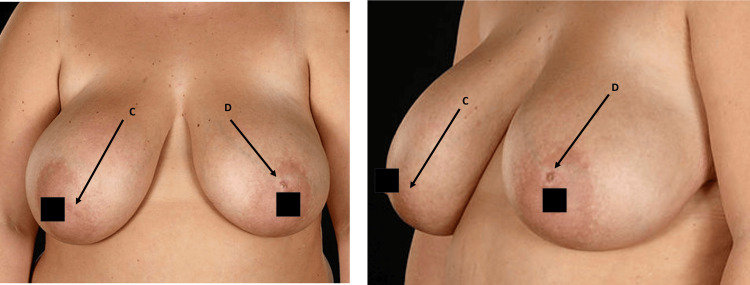
Preoperative images with right and left breast fistula as indicated by labels C and D, respectively

The second case involves a 33-year-old female presenting with recurrent swelling and discharge from the left areola complex. She had no major comorbidities but was a smoker with a BMI of 32 kg/m² at initial presentation. The patient initially underwent excision of the mammary fistula with the wound left open in 2019; however, recurrence occurred within three months. During this time, the patient also developed a right-sided mammary fistula. Following weight reduction and smoking cessation, she underwent excision of bilateral mammary duct fistulae combined with bilateral reduction mammoplasty in 2022. At eight months of follow-up, she remained free of disease recurrence, and cosmetic outcomes were satisfactory. Figure 3 shows preoperative images of a left breast fistula. 

**Figure 4:**
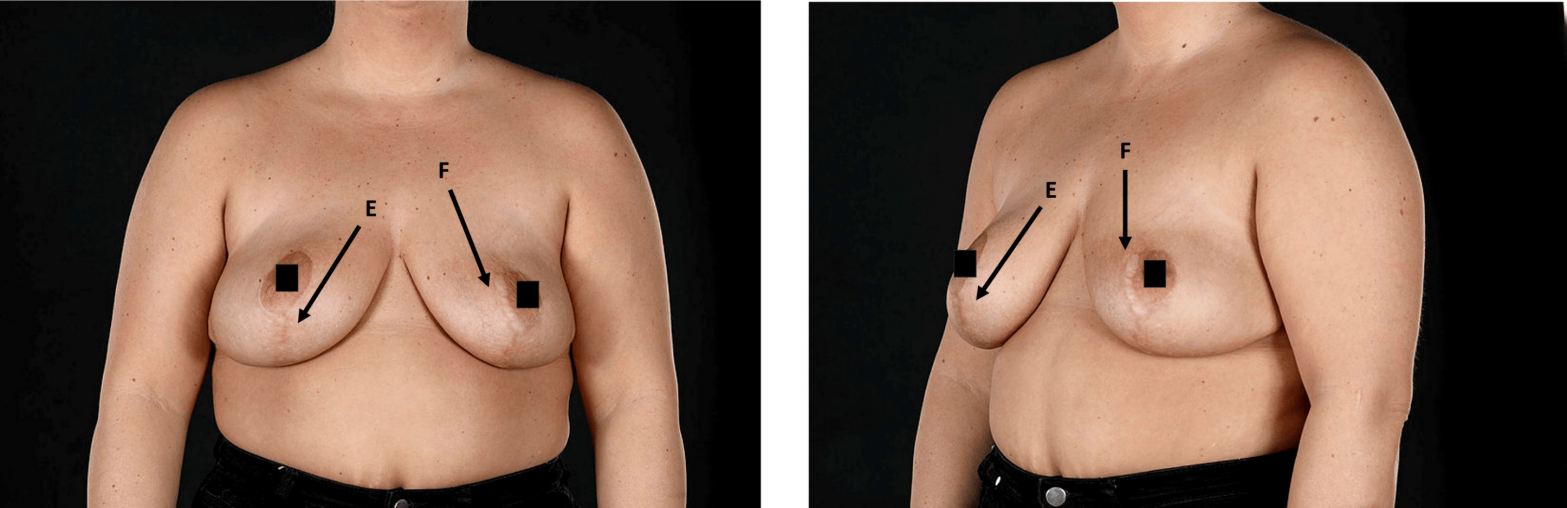
Post-operative images obtained one year after surgery. The scar is indicated by labels E and F.

Figure 4 shows postoperative outcomes one year post surgical intervention. 

Histopathological examination in both cases demonstrated a fistulous tract lined by granulation tissue with features of acute inflammation. There were no granulomas identified, and no evidence of malignancy was observed.

These cases illustrate that, in selected patients with recurrent mammary fistulae unresponsive to conventional interventions, a more radical oncoplastic approach combining fistula excision with reduction mammoplasty can achieve durable disease control while preserving aesthetic outcomes. They highlight the importance of patient optimisation, including management of modifiable risk factors, prior to definitive surgery. 

## Discussion

Chronic breast abscesses are a challenging clinical entity that can significantly affect both the physiological and psychological well-being of patients [3,4]. The clinical presentation of chronic breast abscesses is variable, ranging from localised breast pain to chronically discharging sinuses, which can result in substantial morbidity [5,6]. The chronicity of the disease, coupled with recurrent symptoms, can severely impact daily functioning and quality of life. Importantly, the diagnosis of a chronic breast abscess should only be confirmed once malignancy has been excluded [6], as the clinical and radiological features may mimic inflammatory breast carcinoma or other malignant processes.

Conventional management of chronic breast abscesses primarily involves medical therapy. Antibiotics remain the first-line treatment, often supplemented with corticosteroids when granulomatous mastitis is suspected as the underlying aetiology [6]. Initial short courses of corticosteroids can be escalated to longer or continuous regimens in cases resistant to standard therapy. For patients with suspected ductal ectasia or periductal mastitis, conservative management can be trialled; however, surgical intervention in the form of excision of the affected duct remains the standard of care when symptoms persist [2]. Despite these measures, recurrence rates remain high [5-7], and management of recurrent disease is poorly standardised, with no universally accepted approach currently described in the literature.

Surgical management of chronic breast abscesses carries its own set of challenges. Traditional excisional techniques can result in complications such as skin retraction, scarring, breast deformity, and the potential requirement for further interventions, all of which may exacerbate the psychological and physical burden on patients. Consequently, there is an increasing recognition of the need to integrate oncoplastic principles into the management of benign breast disease to optimise both functional and cosmetic outcomes. Oncoplastic surgery, originally developed for breast cancer management, combines oncologic resection principles with reconstructive techniques to preserve or enhance breast aesthetics. Its application in benign disease is emerging, particularly in cases where repeated interventions or extensive tissue excision are necessary.

**Figure 5:**
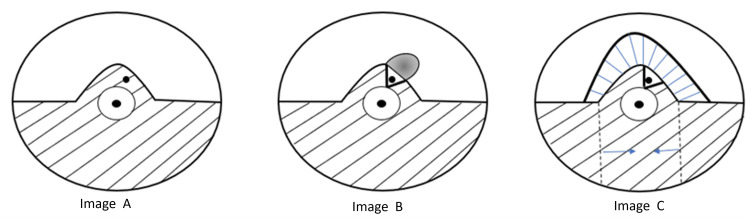
Diagram showing the principles of the surgical intervention Image A: Location of fistula. Image B: Extensive central excision was undertaken, and the breast was remodelled. A lacrimal probe was used to identify unhealthy ducts. Image C: A bipedicle approach was utilised to avoid vascular compromise to the nipple. Defect and skin closed. This figure has been created by the authors.

In the two cases presented, we employed a level 2 oncoplastic approach with bipedicle mammoplasty and contralateral symmetrisation. The surgical technique was guided by visual and tactile feedback to identify diseased tissue accurately, ensuring that all affected ducts and fistulae were excised while preserving healthy tissue to achieve optimal cosmetic outcomes. Figure 5 illustrates the operative steps: Image A shows the localisation of the fistula; Image B demonstrates extensive central excision with remodelling of the breast and use of a lacrimal probe to identify unhealthy ducts; Image C illustrates the bipedicle approach to preserve vascular supply to the nipple, followed by closure of the defect and overlying skin. This method allowed definitive treatment of chronic disease while maintaining breast contour, symmetry, and overall aesthetics.

Various surgical techniques have been described in the literature for chronic breast abscesses, each with variable success and recurrence rates. In peri-areolar abscesses, it is widely accepted that dilated lactiferous ducts should be excised to minimise the risk of recurrence [7-9]. The Hadfield procedure utilises a circumferential peri-areolar incision with fistula excision and is often considered in simple or isolated cases. However, recurrence rates following this technique remain notable, particularly in complex or multifocal disease. The Beechy-Newman approach involves fistulectomy with wide excision of dilated ducts and healing by secondary intention [4]. While effective in some patients, secondary intention healing may result in significant scarring and breast deformity. The variability in surgical technique and outcomes underscores the need for a more standardised, reproducible approach that balances disease eradication with cosmetic preservation.

The use of oncoplastic mammoplasty for benign breast conditions, such as chronic abscesses and periductal mastitis, is not extensively documented. Our cases demonstrate that this approach can achieve durable disease control with low recurrence while also preserving or enhancing cosmetic outcomes. Both patients experienced complete resolution of symptoms, no recurrence at follow-up, and high satisfaction with breast aesthetics, highlighting the dual benefit of functional and psychological improvement. Furthermore, patient optimisation, including smoking cessation, weight management, and careful preoperative planning, played a crucial role in improving surgical outcomes and minimising complications.

These findings suggest that oncoplastic principles, traditionally reserved for malignant disease, may be effectively adapted for selected benign conditions that are refractory to conventional treatment. By allowing excision of diseased tissue with preservation of vascular supply, symmetry, and breast contour, mammoplasty offers a viable solution for patients with recurrent or complex chronic breast abscesses. Importantly, this approach addresses both the physical and psychological sequelae associated with chronic disease, supporting improved patient quality of life.

While our experience is limited to two cases, the outcomes observed support the rationale for further investigation. Prospective studies or randomised controlled trials are warranted to evaluate the broader applicability, long-term outcomes, and recurrence rates associated with oncoplastic interventions in benign breast disease. Such studies would also facilitate the development of standardised protocols, allowing surgeons to select optimal techniques tailored to disease severity and patient-specific factors.

Chronic breast abscesses pose a considerable therapeutic challenge, particularly when conservative and traditional surgical interventions fail. Oncoplastic mammoplasty provides a promising alternative, combining definitive disease control with superior aesthetic outcomes and improved patient well-being. The cases presented underscore the potential of this approach to fill a gap in current management strategies and highlight the need for further research to establish its role in standard clinical practice.

## Conclusions

Problematic nipple discharge and recurrent areolar abscesses can significantly compromise both psychological and physiological wellbeing. When conservative and conventional surgical measures fail, mammoplasty offers a potential definitive treatment. The cases presented demonstrate that, when applied appropriately, mammoplasty not only provides durable disease control but also achieves favourable cosmetic and psychological outcomes by incorporating oncoplastic principles. This approach should be considered a viable option for selected patients with recurrent breast abscesses unresponsive to standard management.
